# Sharp decline of malaria cases in the Burie Zuria, Dembia, and Mecha districts, Amhara Region, Ethiopia, 2012–2014: descriptive analysis of surveillance data

**DOI:** 10.1186/s12936-016-1133-9

**Published:** 2016-02-19

**Authors:** Yumi Toyama, Masaki Ota, Getinet Molla, Belay Bezabih Beyene

**Affiliations:** Japan Anti-Tuberculosis Association, 3-2-24 Matsuyama, Kiyose City, Tokyo 204-8533 Japan; Japan International Cooperation Agency Amhara Region Surveillance and Response Project, P.O. Box 2201, Bahir Dar, Amhara Region Ethiopia; Amhara National Regional State Health Bureau, P.O. Box 495, Bahir Dar, Amhara Region Ethiopia

**Keywords:** Malaria, Surveillance, Epidemiology, Ethiopia

## Abstract

**Background:**

In the Amhara Region of Ethiopia, a steep decline of malaria cases was seen in early 2014. This study verified the decrease of the malaria cases along with the positivity rates among acute febrile illness patients, from late 2012 through 2014 in selected districts of the Amhara Region of Federal Republic of Ethiopia.

**Methods:**

Descriptive epidemiological analysis was conducted on the routine malaria surveillance data from the World Health Organization epidemiological week 28 of 2012 to week 52 of 2014 in three districts: Burie Zuria, Dembia and Mecha, the Amhara Region in Ethiopia. The authors visited the three district health offices, and health centres, when necessary, and collected the surveillance data on malaria for that period.

**Results:**

The study found that the malaria cases, along with the positivity rates, decreased from late 2012 to early 2014 in all three districts. Though the situation had slightly reverted in late 2014, the numbers of cases were much smaller than in late 2012 in all three districts. Despite the different diagnostic techniques used at health centres (malaria microscopy) and health posts (rapid diagnostic tests), moderate to high correlations were found, suggesting that the trends were real and not caused by a defect in the reagent, differences in the technicians’ skills for microscopy, or a change of the health workers’ attitudes toward cases with acute febrile illness. The decrease in malaria cases in early 2014 may have resulted from successful implementation of the three pillars of malaria control—case management, indoor residual spraying and insecticide-treated nets—in the districts where a high percentage of households were protected by indoor residual spraying and/or insecticide-treated nets.

**Conclusion:**

While the current efforts for malaria control should be strengthened and maintained, the review of malaria surveillance data should also be used to verify the malaria trend in the region.

## Background

Malaria remains a major public concern in Ethiopia, where 68 % of the population is estimated to be at risk for malarial infection each year [1]. Aiming to eliminate malaria by 2015 in specific geographical areas with historically low malaria transmission rates as well as near-zero deaths caused by malaria, the Federal Ministry of Health of the Federal Republic of Ethiopia has initiated a massive scale-up of malaria control, including case diagnosis and management, distribution of long-lasting insecticidal nets (LLIN) and indoor residual spraying (IRS) in 2005 [2]. Several studies have been conducted to document declines of malaria cases in the country. While some argue that declining trends in malaria incidence were already seen before the intervention [3–5], a study conducted in four regions found that inpatient malaria cases in children under 5 years of age declined by 73 % between 2001 and 2007 [6]. At the aggregate level, analysis using the national integrated disease surveillance and response system showed that reported malaria inpatient admissions and deaths declined twofold to threefold between 2005 and 2009, though there was no clear declining trend in the total number or number of clinical and laboratory-confirmed malaria outpatients in the same period [7]. On the other hand, a recent national malaria indicator survey showed a small increase in malaria prevalence in areas at altitudes below 2000 m, from 0.9 % in 2007 to 1.3 % in 2011 [2].

The Japan International Cooperation Agency (JICA) Amhara Region Surveillance and Response Project has been working on strengthening the communicable disease surveillance and response in 22 districts in three zones of the Amhara Region since 2008. The Amhara region is the second most populous region in Ethiopia with a population of 20 million people, accounting for almost a quarter of the total population. Strengthening surveillance and response system is a priority in the region where 75 % of the population is at risk of malaria [8]. In June 2014, a dramatic decrease was seen in the number of malaria cases as well as the positivity rates of microscopy of blood films and rapid diagnostic tests (RDTs) conducted for patients with acute febrile illness in the surveillance data. The total number of malaria cases and the positivity rates of the region declined by 43 and 29 %, respectively, in the period from July 2013 to June 2014 compared to the preceding 12-month period.

There are some concerns about the quality of surveillance data and that of laboratory diagnosis, particularly at the health centre (HC) level, where blood film with microscopy is employed with limited quality control and assurance. Malaria surveillance data are filled on a designated forms and sent from HCs to district health offices. Health posts (HPs) send the forms to the nearest HC physically or transfer the data verbally during a cell phone call, then the data are forwarded to the district health offices. In Ethiopia, malaria is normally diagnosed using microscopy at hospitals and HCs that have laboratories and with RDTs (CareStart™ Malaria HRP-2/pLDH (Pf/PAN) combo test, Access Bio, Inc., USA) of *Plasmodium falciparum* and/or other *Plasmodium* species, including *Plasmodium vivax*, at HPs and HCs without laboratories. While an external quality assessment (EQA) scheme for malaria microscopy has gradually been implemented across Ethiopia [9], significant gaps in malaria laboratory diagnosis at health facilities were observed not only in laboratory infrastructure, equipment, materials, and supply chains, but also in the human resource capacities and supporting mechanisms [10]. Furthermore, a recent study conducted at health facilities in the North Gondar Zone of the Amhara Region identified a relatively high false negative rate (16.3 %) for malaria microscopy as well as misclassification of species [11]. Therefore, this identification of the decrease of malaria cases needs to be reviewed at the district level to make sure the decreases are real. The aim of this study was to review the malaria surveillance data and to verify the decrease of the reported malaria cases and in positivity rates in selected districts of the Amhara region in Ethiopia.

## Methods

### Case definition for malaria cases

A case was defined as a person diagnosed with laboratory confirmation by malaria microscopy or RDT at the HC or HP of Burie Zuria, Dembia, and Mecha districts from the week 28 of 2012 to the week 52 of 2014.

### Study design and selection of sites

This is a descriptive epidemiological analysis of the routine malaria surveillance data from the World Health Organization epidemiological week 28 of 2012 to week 52 of 2014 collected from three districts: Burie Zuria, Dembia and Mecha. The three districts were selected because they had steeper declines in the numbers of test-confirmed cases and in the positivity rates than other districts. In these districts, malaria follows a seasonal pattern with biannual peaks from September to December and April to May [12]. While the transmission patterns depend on rainfall and population movement, areas at altitudes below 2000 m are considered to be malarious [2]. Both *P. vivax* and *P. falciparum* are prevalent in the areas [12, 13]. Burie Zuria is one of the districts in the West Gojam Zone with a population of 165,000 people and with altitudes ranging from 713 to 2604 m above sea level. There are five HCs and 19 HPs reporting the cases in the district. Dembia is one of the districts in the North Gondar Zone with a population of 306,000 people and with altitudes from 1750 to 2100 m above sea level. There are 10 HCs and 40 HPs in the district. One HC in this district used RDTs instead of microscopy from week 28th of 2013 to 52nd of 2014 due to the unavailability of a laboratory technician at the HC. Mecha district is located in the West Gojam Zone with a population of 330,000 people and the altitude ranges from 1900 to 3200 m above sea level. There are 10 HCs and 40 HPs.

### Data collection, entry and statistical analysis

The three district health offices in the three selected districts were visited quarterly in the period from July 2014 to January 2015 to collect the surveillance data, including the number of malaria cases by species of malaria, reporting week, and reporting unit (HC or HP). When disaggregated data were not available at the district health offices, the authors also visited HCs that were accessible by car in the districts to obtain the disaggregated data from the reporting unit. The HCs of Dembia and Mecha were visited every quarter and the HCs of Burie Zuria half-yearly to monitor the quality of the surveillance data, including crosschecks of surveillance reports with laboratory data. Discussion sessions were held with the health care workers at the HC level to explore possible changes in their attitudes with regard to referring patients with acute febrile illness to the laboratory. Because, at the field level, the sensitivity and specificity of RDTs are generally much higher than for smear microscopy [14, 15], the malaria case data were collected by the reporting unit. Then the trend of the positivity rates and proportions of *P. vivax* were compared between the HCs (where smear microscopy is employed) and HPs (where RDTs are used). Furthermore, the routine surveillance data of the three district was compared with the data collected in our study. Data were doubly entered in MS Excel 2003 (Microsoft Corp., Seattle, USA) and discrepancies were corrected against the original data sheet. Statistical analysis was conducted using R software (The R Project). Holm’s pairwise *t* test was employed to do multiple comparisons of the numbers of malaria cases per week among semesters. Pearson’s correlation analysis was conducted for the weekly average positivity rate and proportion of *P.vivax* at HCs against those at HPs of each district for the total of 129 weeks from 28th week of 2012 to the end of 2014. The same analysis was conducted for one HC in Dembia district where RDTs are used and the other nine HCs where microscopy is employed for the total of 77 weeks from 28th week of 2013 to the end of 2014. A probability level of p < 0.05 was considered statistically significant.

### Ethical considerations

The authors obtained ethical clearance from the Research Ethics Committees of the Amhara National Regional State Health Bureau, Ethiopia and the Research Institute of Tuberculosis, Tokyo, Japan.

## Results

The authors obtained the disaggregated malaria surveillance data from all the HCs and HPs of Burie Zuria, Dembia and Mecha for the study period except that in Burie Zuria from 43rd to 52nd week of 2014, in Dembia from 28th week of 2012 to 27th week of 2013 and 41st to 45th week of 2014, in Mecha 42nd to 52nd week of 2014, in which only aggregated data for all the HPs of each cluster were available, because of the change in the way of collecting surveillance data. The routine surveillance data aggregated for districts were available from 16th week of 2013 to 52nd week of 2014.

**Fig. 1:**
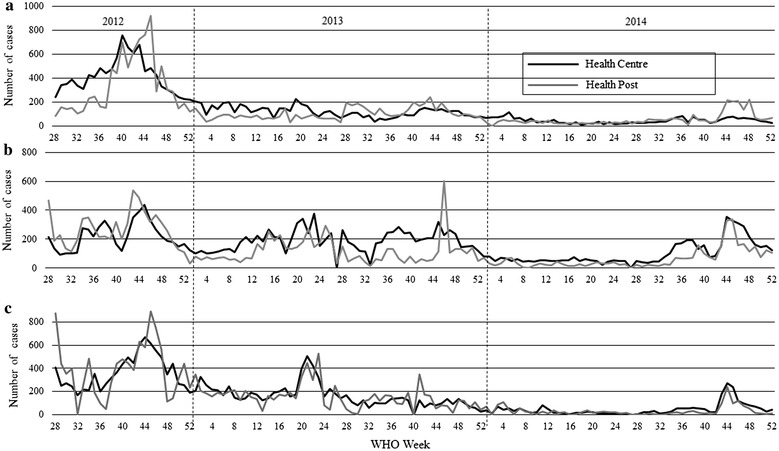
The numbers of malaria cases in three districts by week, Amhara Region, Ethiopia, 2012–2014. **a** Burie Zuria; **b** Dembia; **c** Mecha

 Figure 1a–c shows the total numbers of laboratory-confirmed malaria cases in the Burie Zuria, Dembia, and Mecha districts from the 28th WHO epidemiological week of 2012 to the 52nd of 2014. The trend of the numbers of cases was similar between HCs and HPs in all three districts. In general, there were seasonal changes in the numbers of cases in the three districts, though in Burie Zuria the changes were less prominent than in the other districts. Relatively lower numbers of malaria cases were seen in the three districts in early 2014, particularly before the 32nd week; however, after the 41st week the numbers of cases were higher than during the first 41 weeks of 2014 in all three districts.

**Fig. 2:**
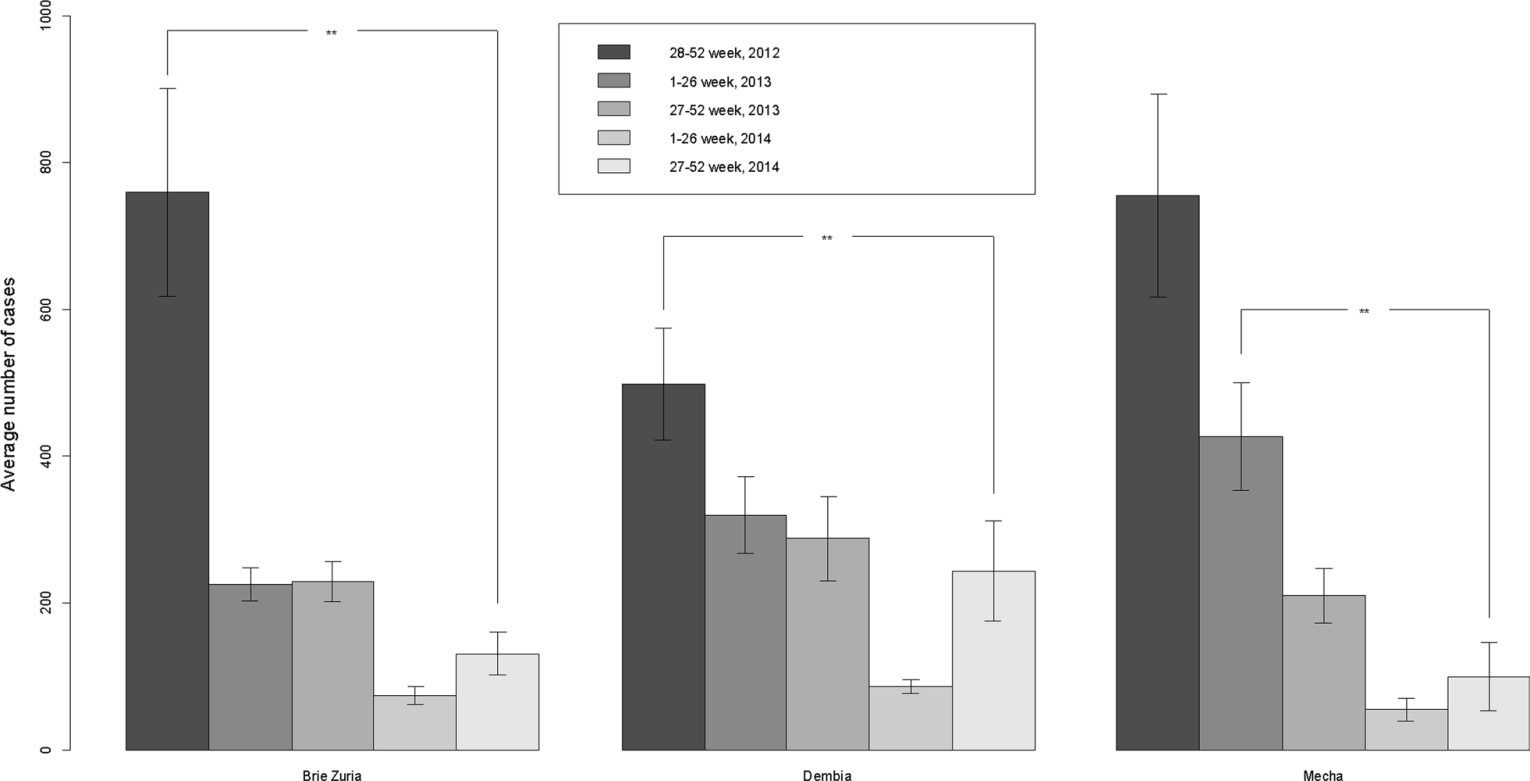
Average numbers of confirmed malaria cases by semester in three districts, Amhara Region, Ethiopia, 2012 (The 2012 data only include the 28th to 52nd epidemiological weeks) to 2014. **p value < 0.01. *Error bars* indicate 95 % confidence intervals

The average numbers of malaria cases per week in the three districts in the first semester of 2014 sharply declined compared with the previous semesters (Fig. 2). The average numbers of cases were significantly lower in the second semester of 2014 than in the second semester of 2012 in all three districts.

**Fig. 3:**
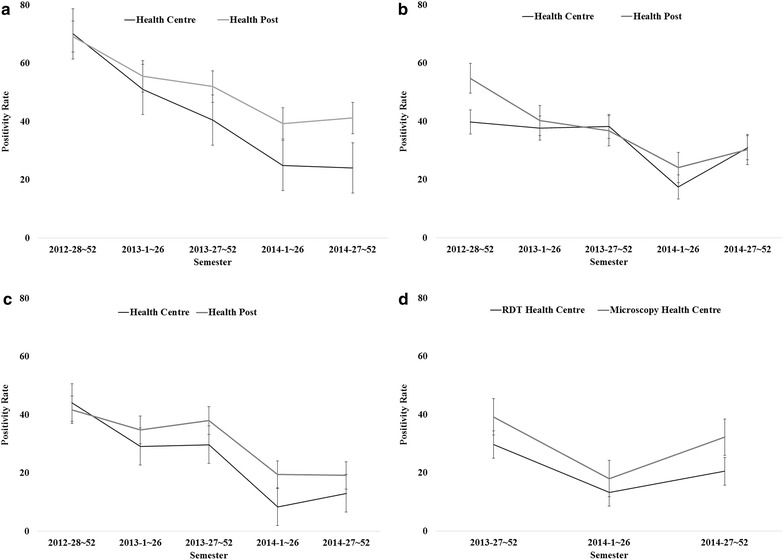
Trends of positivity rates for malaria tests in three districts, Amhara Region, Ethiopia, 2012–2014. **a** Burie Zuria; **b** Dembia; **c** Mecha; **d** Dembia (HCs)

Figure 3a–c shows the average positivity rates of laboratory tests for malaria of the three districts reported by HPs and HCs by semester from late 2012 to late 2014. The trend of the positivity rates of the three districts are parallel at HPs and HCs and declined towards late 2014, though in Dembia the positivity rate slightly reverted from early to late 2014. Correlation analyses revealed that the weekly average positivity rates at HCs had statistically significant and moderate to high correlation with those at HPs in Burie Zuria [R = 0.72 (95 % CI 0.62–0.79)], Dembia [R = 0.47 (95 % CI 0.33–0.60)] and Mecha [R = 0.70 (95 % CI 0.60–0.78)]. Figure 3d shows the same analysis between one HC in Dembia district where RDTs are used and the other 9 HCs where microscopy is employed from late 2013 to late 2014. A statistically significant and moderate correlation was found in the weekly average positivity rates between two types of HCs [R = 0.50 (95 % CI 0.32–0.66)].

**Fig. 4:**
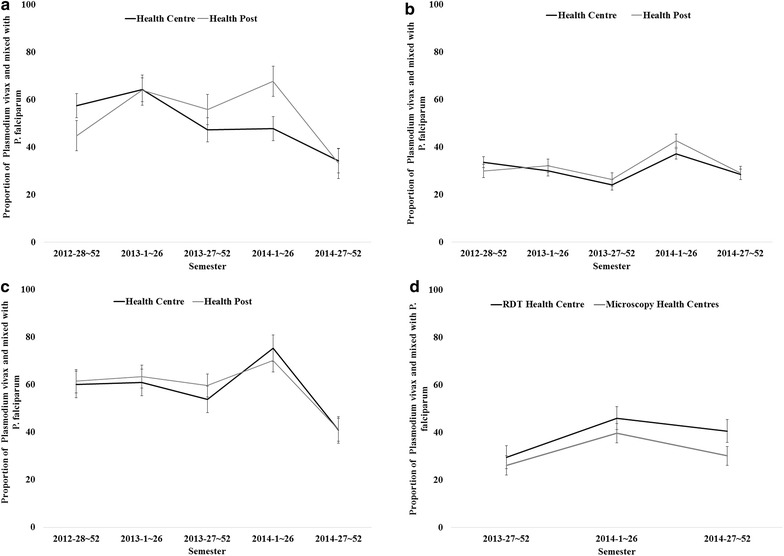
Trends of proportion of *Plasmodium vivax* malaria in three districts, Amhara Region, Ethiopia, 2012–2014. **a** Burie Zuria; **b** Dembia; **c** Mecha; **d** Dembia (HCs)

Figure 4a–c shows the average proportions of infections with only *P. vivax* or mixed species among all positive cases of the three districts by HCs and HPs by semester from late 2012 to late 2014. The trend of the proportions of *P. vivax* were parallel between HCs and HPs in the three districts, except in the 2nd semester of 2013 and 1st semester of 2014 in Burie Zuria, in which the proportion of *P. vivax* and mixed species at HPs was higher than at HCs. Correlation analyses revealed that the weekly average proportions of *P. vivax* plus mixed species at HCs had statistically significant and moderate correlation with those at HPs in Burie Zuria [R = 0.42 (95 % CI 0.26–0.55)], Dembia [R = 0.40 (95 % CI 0.24–0.53)], and Mecha [R = 0.63 (95 % CI 0.51–0.72)]. Figure 4d shows the same analysis between one HC in Dembia district where RDTs are used and the other 9 HCs where microscopy is employed from late 2013 to late 2014. Though it was not statistically significant (p = 0.057), a moderate correlation was found in the weekly average proportions of *P. vivax* plus mixed species between the two types of HCs [R = 0.30 (95 % CI 0.01–0.56)].

Table 1 shows the comparisons of the numbers of confirmed malaria cases collected in our study with the routine surveillance data by semester from 16th week of 2013 to 52nd week of 2014. While the difference between the routine and study data has been decreased to almost none in the 2nd semester of 2014 in Burie Zuria, the differences increased to 7 % in the same semester in Dembia. Even bigger discrepancies were observed in Mecha: 28 % less cases were found in the study data in 2nd semester of 2014.

**Table 1:** Comparisons of numbers of confirmed malaria cases in three district, Amhara Region, Ethiopia, 2013^a^–2014

District	2013—16 to 26	2013—27 to 52	2014—1 to 26	2014—27 to 52
*Burie Zuria*				
Study data	2384	6113	1924	3399
Surveillance data	3577	6234	1819	3413
Difference	−1193	−121	105	−14
Difference (%) divided by study data	−50	−2	5	0
*Dembia*				
Study data	4720	7942	2238	6329
Surveillance data	4692	7728	2378	6802
Difference	28	214	−140	−473
Difference (%) divided by study data	1	3	−6	−7
*Mecha*				
Study data	5607	5887	1438	2595
Surveillance data	5647	4697	1136	3331
Difference	−40	1190	302	−736
Difference (%) divided by study data	−1	20	21	−28

^a^The 2013 data only include the weeks from 16th to 52nd epidemiological weeks

The investigators found no changes in the attitudes of the clinicians at the health centres toward ordering blood film tests for outpatients with acute febrile illness during the monitoring visits.

The proportion of health facilities that timely reported the malaria surveillance data has improved from 93.7 % (95 % CI 92.3–95.0) on average in the second semester of 2013 to 99.1 % (95 % CI 98.7–99.6]) on average in the second semester of 2014.

## Discussion

This is a review for the malaria surveillance data of three districts in the Amhara Region of north western Ethiopia. The investigation verified that both the numbers of malaria cases and the positivity rates decreased from late 2012 to 2014 in all three districts. The malaria data were analysed separately for HCs and HPs and moderate to high correlations were found between the two in terms of the positivity rates and the proportions of *P. vivax* plus mixed species infections. This suggested that the declines in the number of malaria cases at HCs were real, not caused by a defect in the reagent, differences in technicians’ skills for microscopy, or changes of health workers’ attitudes toward cases with acute febrile illness. If any of these had been the case, a correlation in the positivity rates between HCs, where microscopy was used, and HPs, where RDTs were used, would be less likely. The fact that the species identification of *P. vivax* at HCs correlated with species identification done using RDTs at HPs suggested that the diagnosis of malaria at the HCs did in fact reflect a real trend, though there may be misdiagnosis with malaria microscopy on a case-by-case bases reported previously [11].

Furthermore, the declines of malaria cases were not caused by the declines of the numbers of health facilities, either, in the three districts that involved in the malaria surveillance system, since the proportion of the HCs and HPs that timely reported weekly surveillance data in the districts have increased between late 2013 and late 2014.

The study findings are consistent with the recent malaria report by the Federal Ministry of Health of the Federal Republic of Ethiopia that there was a steep decrease between September 2012 and August 2013 in Amhara Region along with other four regions (Southern Nations Nationalities and People’s, Oromia, Tigray and Afar) [8].

Although the sensitivity of RDTs for detecting *P. falciparum* is higher than for *P. vivax* [16], increased proportion of *P. vivax* in Dembia district in 2014 compared with the past 2 years could support decreasing trend of malaria transmission in the area. The shift from *P.falciparum* to *P. vivax* are seen in a different study conducted at one of the HCs in Dembia [13], aggregated level in Ethiopia [7] and other countries in the process towards elimination [17, 18].

While lower cases of malaria were observed in the first semester of 2014 in the study districts, seasonal rainfall in this period, including a peak malaria period from April to May [12], was normal as in the past in the Amhara region [19].

The strength and limitations were the following. Since the authors directly obtained the malaria surveillance data from district health offices and from HCs and almost all the HCs of the three districts were routinely visited with the malaria data being crosschecked with laboratory register, the data were considered validated and accurate. One of the limitations is that in Burie Zuria, one HC’s data were missing from week 28th of 2012 to week 27th of 2013, which led about 50 % less cases in the study collected data than in the routine surveillance data in the 1st semester of 2013. However, the decrease of the numbers of malaria cases in the early and late 2014 compared in the late 2012 is still valid in Burie Zuria. In Mecha, the discrepancy between the routinely collected data and study collected data in the 2nd semester of 2014 may have been due to the change of reporting flow, in which three HPs have been assigned to report to different HCs within the district, and this may have led them to delay the reporting and delayed data may have been added to the reports of the later weeks. Although the routine surveillance data show that malaria cases were reported in Dembia and Mecha in the 27th week of 2014, the starting week of Ethiopian fiscal year, the authors were not able to verify the cases caused by the lack of the documents, probably due to the staff turnover and/or failing to make a report on time in the weeks. This is a review of the public surveillance data and it was not possible to segregate new and recrudescent infections, because the reporting form does not require the health staff to report separately. Also, it is possible that a same person may have been doubly counted as malaria cases when the person attends first an HP and then proceeds to an HC, referred by the HP, because the HC and HP are only require to report the number of confirmed malaria cases.

A possible reason for the decrease in the number of malaria cases in the Amhara Region toward early 2014 is the successful implementation of the three pillars of malaria control: case management, IRS and ITNs [9]. As a country, the population protected by any vector control was more than 60 % in 2012 in Ethiopia, which is the best performing situation compared to other Sub-Saharan African countries [1]. The recent malaria indicator survey performed from October to December 2011 found that Amhara region had a higher percentage of households protected by LLIN and/or IRS (86.3 %) than the other regions of Ethiopia [2], having at least one mosquito net [20] and living in a house treated with IRS [12], which are associated with a lower risk of malaria prevalence in Ethiopia. Thus, these findings may support the impacts of malaria control efforts in Amhara region.

## Conclusion

Since the current malaria control strategy has been quite effective in reducing the burden of malaria in the Amhara Region, the Amhara Regional Health Bureau should strengthen and maintain the current efforts at case management, IRS and ITNs. In addition, to monitor the trend of malaria cases as well as positivity rates, the malaria surveillance system should be strengthened and maintained. The malaria surveillance data should also be periodically reviewed to make sure the decreasing trend continues.

## Abbreviations

EQA: external quality assessment
HC: health centre
HP: health post
IRS: indoor residual spraying
JICA: Japan International Cooperation Agency
LLIN: long-lasting insecticidal nets
RDTs: rapid diagnostic tests
